# A WHO-HPH operational program versus usual routines for implementing clinical health promotion: an RCT in health promoting hospitals (HPH)

**DOI:** 10.1186/s13012-018-0848-0

**Published:** 2018-12-22

**Authors:** Jeff Kirk Svane, Shu-Ti Chiou, Oliver Groene, Milena Kalvachova, Mirna Zagrajski Brkić, Isao Fukuba, Tiiu Härm, Jerneja Farkas, Yen Ang, Mikkel Østerheden Andersen, Hanne Tønnesen

**Affiliations:** 1 Clinical Health Promotion Centre, WHO-CC, Bispebjerg and Frederiksberg Hospital, Copenhagen University Hospitals, Nordre Fasanvej 57, Build. 14, Entr. 5, 2nd fl, 2000 Frederiksberg, Denmark; 2 School of Medicine, National Yang-Ming University, Taipei, Taiwan; 3 Cheng Hsin General Hospital, Taipei, Taiwan; 4 OptiMedis AG, Hamburg, Germany; 5 Department of Health Services Research and Policy, London School of Hygiene & Tropical Medicine, London, UK; 6 Health Services Quality Department, Ministry of Health, Prague, Czech Republic; 7 General hospital “Dr. Tomislav Bardek”, Koprivnica, Županija Koprivničko-križevačka Croatia; 8 Saitama Cooperative Hospital, Kawaguchi, Saitama Japan; 9 National Institute for Health Development;, Tallin, Estonia; 10 National Institute of Public Health, Ljubljana, Slovenia; 11 Penang Adventist Hospital, Penang, Malaysia; 12 Sector for Spine Surgery and Research, Lillebaelt Hospital, Middelfart, Denmark; 13 Clinical Health Promotion Centre, WHO-CC, Health Sciences, Lund University, Lund, Sweden

**Keywords:** Strategic implementation, Fast-track implementation, Quality improvement, Clinical health promotion, Health promoting hospitals, Lifestyle risk, Patients, Hospital staff

## Abstract

**Background:**

Implementation of clinical health promotion (CHP) aiming at better health gain is slow despite its effect. CHP focuses on potentially modifiable lifestyle risks such as smoking, alcohol, diet, and physical inactivity. An operational program was created to improve implementation. It included patients, staff, and the organization, and it combined existing standards, indicators, documentation models, a performance recognition process, and a fast-track implementation model.

The aim of this study was to evaluate if the operational program improved implementation of CHP in clinical hospital departments, as measured by health status of patients and staff, frequency of CHP service delivery, and standards compliance.

**Methods:**

Forty-eight hospital departments were recruited via open call and stratified by country. Departments were assigned to the operational program (intervention) or usual routine (control group). Data for analyses included 36 of these departments and their 5285 patients (median 147 per department; range 29–201), 2529 staff members (70; 10–393), 1750 medical records (50; 50–50), and standards compliance assessments.

Follow-up was measured after 1 year. The outcomes were health status, service delivery, and standards compliance.

**Results:**

No health differences between groups were found, but the intervention group had higher identification of lifestyle risk (81% versus 60%, *p* < 0.01), related information/short intervention and intensive intervention (54% versus 39%, *p* < 0.01 and 43% versus 25%, *p* < 0.01, respectively), and standards compliance (95% versus 80%, *p* = 0.02).

**Conclusions:**

The operational program improved implementation by way of lifestyle risk identification, CHP service delivery, and standards compliance. The unknown health effects, the bias, and the limitations should be considered in implementation efforts and further studies.

**Trial registration:**

ClinicalTrials.gov: NCT01563575. Registered 27 March 2012. https://clinicaltrials.gov/ct2/show/NCT01563575

**Electronic supplementary material:**

The online version of this article (10.1186/s13012-018-0848-0) contains supplementary material, which is available to authorized users.

## Background

Turning evidence into practice in healthcare often takes decades [1–3]. Slow implementation also occurs with patient-centered activities to modify lifestyle risk factors [4], such as clinical health promotion (CHP) aiming at better health gain for patients, staff, and communities.

A sub-type of health promotion [5–7], CHP covers patient-enablement, disease prevention, health promotion, and rehabilitation, which takes place within patient pathways [8, 9]. CHP relies on counseling [10–13] where clinical staff support patients to control and improve both health and modifiable determinants thereof [14, 15], such as daily smoking, risky alcohol drinking, poor nutrition, physical inactivity, and other lifestyle risks [8]. On short term within pathways, CHP has been shown to improve treatment results and prognoses in surgery [16–19], obstetrics [20–22], internal medicine [23–27], and psychiatry [28]. It is also cost-effective [29] and well-received by patients [30–32]. On long term, it can contribute to better public health [16, 33]. Even so, however, CHP is rarely implemented [33].

Furthering implementation of CHP, and of the World Health Organization (WHO) concepts, values, strategies, and standards of health promotion in general, into the organizational structure and culture of hospitals and health services is the aim of the WHO-initiated International Network of Health Promoting Hospitals and Health Services (HPH) [34, 35].

To support the attainment of this goal, a package of validated tools and a recognition of performance (RP) were recently developed by WHO and HPH. The package of tools included five WHO standards with related indicators [34, 36, 37] that were developed according to the International Society for Quality in Health Care (ISQUA) criteria [38], and two HPH documentation models [39, 40]. The RP used HPH certifications recognizing fulfillment of the five WHO standards [41].

To speed up implementation, a 1-year, fast-track implementation model for CHP (Fast-IM) was also added [41]. The Fast-IM was data-driven and used resources related to strategic implementation of evidence [3, 42–49] as well as general quality improvement tools such as the Plan-Do-Check-Act (PDCA) cycle [50]. The Fast-IM aims to set 1-year implementation goals for the individual organization based on own local data identifying important implementation gaps. The Fast-IM incorporates adjustable quality plans with clear, measurable 3-month milestones and is driven by the data [41].

Combined, the package of tools, the RP, and the Fast-IM were evaluated as a WHO-HPH operational program versus usual routines for implementation of CHP. Specifically, the evaluation focused on the operational program’s ability to potentially improve dosage, quality, and fidelity of implementation by way of risk identification, CHP service delivery, and standards compliance, and by this route, possibly, improve the health of patients and staff [41].

### Aim

The aim of this study was to evaluate if the operational program improved implementation of CHP in clinical hospital departments. This was measured by health status of patients and staff and by implementation process in terms of frequency of CHP service delivery and standards compliance.

## Methods

### Participants

For our randomized controlled trial with the clinical hospital department as the unit of randomization and analysis (i.e. no cluster), we hypothesized that allocation to the operational program (intervention group) would improve health of patients and staff, increase delivery of CHP services to at risk-patients, and improve WHO standards compliance at the department level, compared to the control group departments continuing usual implementation routines. The service delivery and standards compliance outcomes serve as measures of procedural and structural changes in implementation, and the health outcomes serve as a measure of the potential effect of such changes.

Inclusion criteria were clinical hospital departments responsible for treatment. Exclusion criteria were > 1 department from each hospital and palliative departments, nursing homes, pediatric departments, non-hospital clinics, and primary care facilities since the CHP-specific process components were not validated in these settings.

Based on a secondary outcome (standards compliance), we calculated a sample size of 2 × 40 clinical departments, because no studies existed on the primary outcome of health status. The power calculation was based on a previous study [40], which had shown that baseline CHP service deliveries could be expected to reach no more than 40% of the at-risk patients. The minimum relevant difference in service deliveries was 30%, the expected outcome was 70%, power was 80%, and two-sided significance was 5% [41].

**Fig. 1:**
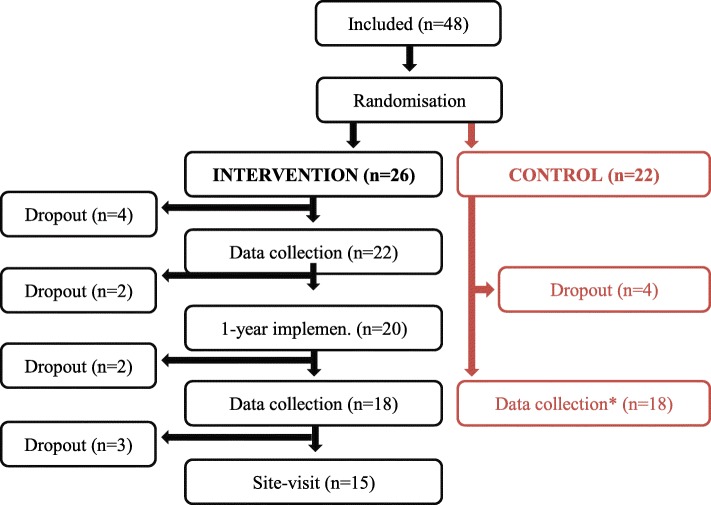
Trial profile

Full inclusion of the 2 × 40 departments, along with the 2-year follow-up, was not obtained before an update and revision of WHO standards in 2016, at which point only 48 departments had been included and randomized (Fig. 1). The 48 included departments that participated were recruited via an open call among HPH member hospitals (Fig. 1). Owing to the commitment of HPH members to use WHO standards, and since the revised WHO standards were markedly different, new centers and already participating centers in the RCT could not be expected to begin to or continue to use the old version.

Of the 48 included departments, 8 (4 in each group) dropped out after allocation. The remaining 40 departments were from 8 countries/regions in Asia and Europe: Taiwan (*n* = 21), Czech Republic (8), Slovenia (3), Croatia (2), Estonia (2), Japan (2), Denmark (1), and Malaysia (1). Of these, 36 (75% of the originally included 48) completed the study. The characteristics at department-level, staff-level, and patient-level are presented in Table 1. The study was registered at ClinicalTrials.gov (NCT01563575).

**Table 1:** Characteristics of the 40 hospital departments that participated and characteristics of the 5285 patients and 2529 staff from the 36/40 clinical departments that submitted data for analyses, presented at the department level as median and range

Hospital departments		Intervention (*n* = 22)	Control (*n* = 18)
Hospital type	Community/general/specialized/university	18/50/9/23%	6/61/11/22%
Ownership	Public/private non-profit/private for-profit	64/27/9%	33/61/6%
Department	Medicine/surgery and obs-gyn/psychiatry	73/18/9%	66/28/6%
Catchment area	Urban/rural/mixed	32/9/59%	61/11/28%
Number of beds	< 200/200–599/> 599	23/36/41%	0/50/50%
HPH member		100%	100%
Patients and staff		Intervention (*n* = 18)	Control (*n* = 18)
Patients (*n* = 5285)		152 (75–201)	142 (29–200)
Age (years) 18–29		8% (0–48%)	5% (0–26%)
30–49		19% (0–56%)	23% (6–66%)
50–69		40% (10–61%)	44% (15–59%)
> 70		33% (0–91%)	28% (0–71%)
Women		52% (28–79%)	55% (32–100%)
BMI		26 (17–44)	25 (16–38)
Daily smoking		10% (0–23%)	10% (2–16%)
Hazardous alcohol drinking		3% (0–13%)	2% (0–8%)
Physical inactivity		60% (15–83%)	66% (32–88%)
Risk of malnutrition		44% (16–72%)	36% (15–60%)
Overweight/obesity		60% (25–92%)	51 (25–86%)
Staff (*n* = 2529)		70 (10–393)	71 (18–223)
Age (years) 18–29		28% (0–66%)	25% (4–52%)
30–49		53% (29–80%)	59% (40–73%)
> 50		19% (0–60%)	16% (0–43%)
Women		80% (49–95%)	85% (64–97%)
BMI		23 (17–34)	23 (17–34)
Daily smoking		5% (0–20%)	8% (0–37%)
Hazardous alcohol drinking		1% (0–9%)	1% (0–12%)
Physical inactivity		73% (24–100%)	79% (27–99%)
Risk of malnutrition		26% (4–60%)	27% (7–47%)
Overweight/obesity		33% (10–75%)	28% (12–52%)

Missing data 0–9%

### Randomization and masking

We randomly allocated departments to undertaking the operational program (intervention group) or to continuing usual implementation routines (control group). An external researcher performed the computerized randomization, using blocks of unknown sizes and stratification by country. Sealed, opaque envelopes concealed allocation numbers from the international research team that enrolled departments. All allocation was video recorded. In view of the nature of the intervention, the participating departments and project staff were aware of their allocation.

### Procedures

All data were collected between October 2012 and October 2016. Each department’s data collection took 1–3 months. The international research team developed and provided project instruction manuals, forms, and templates [41] (see Additional file 1). All translation and provision of information to staff was handled locally. An introduction seminar covering data collection, surveying, and auditing training was held for staff at all participating departments (online or on location) before randomization and project start.

Participating departments assigned data collection tasks to one or more of their own staff according to local needs and resource availability.

#### Intervention group

The intervention group began the operational program [41] immediately after allocation. After 1 year, they repeated the data collection and underwent an external audit including interviews with staff and managers [41, 51].

#### Control group

To reduce the risk of contamination, the control group departments did not measure pre-implementation status but instead waited 1 year. During this wait, they continued their own usual implementation routines (understood as usual management activity—parallel to the clinical term, treatment as usual). These usual implementation procedures presumably varied among participating hospitals, and since all were HPH member hospitals, many worked with the WHO standards already. No data was collected on usual implementation routines in each hospital in advance of the project. After the 1-year wait, the control group departments collected their data (Fig. 1). For convenience, and only after the trial had ended, the control group was also offered the operational program. This offer was accepted by 17/18 control group departments.

#### Data collection (both groups)

The collected data covered patient, staff, and department levels. Validated Short-Form 36 version 2 (SF36v2) health surveys [52, 53] were used to assess patient and staff health. As described in the RCT protocol, each department surveyed 200 consecutive patients or 1 month’s population of patients, depending on which of these two stopping-points were reached first, and all staff currently employed by the department [41]. The eight dimensions of SF36v2 were summarized in physical (PCS) and mental (MCS) component scores for analyses, where a score of 100 represented maximum functionality. For identification of patient lifestyle risks and related CHP service delivery, validated medical record audit tools were used [39, 40] according to the operational program [41]. Here, each department audited 50 consecutive medical records concerning documentation of patient lifestyle risks, using the HPH DATA Model [39], and concerning associated delivery of relevant CHP services, using the HPH DocAct Model [40], as described in the protocol [41]. If data was available in the medical record, e.g., “does the patient smoke daily?”, the auditing staff member would answer “yes” or “no” as relevant, and that would count as documented risk (either positive or negative). If data was unavailable, the staff member would answer “unknown” and that would count as undocumented risk [39].

The same approach was used for auditing of CHP service documentation in the records [40]. CHP services can be categorized as either short interventions (SI) or intensive interventions (II) [54, 55]. SI do not exceed three counseling sessions and/or a total contact time of 1 h [55]. II consist of four or more in-person sessions of 10 min or more each [54, 55]. II are often theory-based, offered by trained staff, include patient education and pharmacological support. While the SI/II categorization [54, 55] was used in the study, the design and contents of each CHP intervention were determined locally.

Compliance with the validated WHO standards [34, 36, 37] was also assessed (Table 2). The WHO standards contain 40 measurable elements within five standard domains [34] (see Table 2). Department performance was recognized with a certificate based on standard compliance (91–100% was gold level).

**Table 2:** The 5 WHO standards for health promotion in hospitals: Compliance to the 40 measurable elements (ME) of the intervention and control group, presented as median and range

Standard	No. of ME	Description	Objective	Intervention group (*n* = 18)	Control group (*n* = 18)	*p*
1 Management policy	9	The organization has a policy for HP. The policy is implemented as part of the overall QM system.	To describe the framework for the organization’s HP activities as an integral part of the QM system.	8 (6–9)	7 (3–9)	
		Measurable elements: 1. Stated aims and mission include HP 2. Minutes of governing body reaffirms agreement within the past year to participate in the WHO HPH Network 3. The current quality and business plans include HP for patients, staff and the community 4. Personnel and functions for the coordination of HP are identified 5. There is an identifiable budget for HP services and materials 6. Operational procedures such as practice guidelines or pathways incorporating HP are available in clinical departments 7. Specific structures and facilities required for HP (including resources, space, equipment) can be identified 8. Data are routinely captured on HP interventions and available to staff for evaluation 9. A programme for quality assessment of HP activities is established				
2 Patient assessment	7	In partnership with patients, staff systematically assess the needs for HP activities.	To support patient treatment, improve prognosis and promote the health and wellbeing of patients.	7 (5–7)	6 (1–7)	
		Measurable elements: 1. Guidelines on how to identify smoking, alcohol consumption, nutritional and psycho-social-economic status are present 2. Guidelines/procedures have been revised within the last year 3. Guidelines are present on how to identify needs for HP for groups of patients (e.g. asthma patients, diabetes patients etc.) 4. The assessment is documented in the patient’s medical record at admission 5. There are guidelines/procedures for reassessing needs at discharge or end of a given intervention 6. Information from referring physician or other relevant sources is available in the patient’s record 7. The patient’s record documents social and cultural background as appropriate				
3 Patient information and intervention	6	Patients receive info on significant factors concerning disease/condition, and HP interventions are established in all pathways.	To ensure patients are informed about activities, empowered in an active partnership and to facilitate integration of HP activities in all pathways.	6 (4–6)	4 (1–6)	
		Measurable elements: 1. Information given to the patient is recorded in the patient’s records 2. Health promotion activities and expected results are documented and evaluated in the records 3. Patient satisfaction assessment of the information given is performed and the results are integrated into the QM system 4. General health information is available 5. Detailed information about high-risk diseases is available 6. Information is available on patient organizations				
4 Promoting a healthy workplace	10	The management establishes conditions for the development of a healthy workplace.	To support the development of a healthy and safe workplace and to support health promotion activities of staff.	10 (7–10)	8 (2–10)	
		Measurable elements: 1. Working conditions comply with national/regional directives and indicators 2. Staff comply with health and safety requirements and all workplace risks are identified 3. New staff receive an introductory training that addresses the hospital’s HP policy 4. Staff in all departments are aware of the content of the organization’s health promotion policy 5. The performance appraisal system and continuing professional development include HP 6. Working practices (procedures and guidelines) are developed by multidisciplinary teams 7. Staff are involved in hospital policy-making, audit and review 8. Policies for awareness on health issues are available for staff 9. Smoking cessation programmes are offered 10. Annual staff surveys are carried out including an assessment of individual behaviour, knowledge on supportive services/policies, and use of supportive seminars				
5 Continuity and cooperation	8	The organization has a planned approach to collaboration with other providers and other institutions and sectors.	To ensure collaboration with relevant providers and initiate partnerships to optimize integration of HP activities in pathways.	8 (7–8)	7 (3–8)	
		Measurable elements: 1. The management board is taking the regional health policy plan into account 2. The management board can provide a list of health and social care providers working in partnership with the hospital 3. The intra- and intersectoral collaboration with others is based on execution of the regional health policy plan 4. There is a written plan for collaboration with partners to improve the patients’ continuity of care 5. Patients/families are given understandable follow-up instructions at out-patient consultation, referral or discharge 6. There is an agreed upon procedure for exchange practices between organizations for all relevant patient information 7. The receiving organization gets a written summary of condition, health needs and interventions already provided 8. If appropriate, a plan for rehabilitation describing roles of the organization/collaborators is documented in the record				
Overall compliance	40			38 (31–40)	32 (12–40)	*0.02*

*HP* (clinical) health promotion, *QM* quality management, overall 3% missing data

### Outcomes

The primary outcome was health status of patients and staff as measured by SF36v2. The secondary outcomes were CHP service delivery to identified at-risk patients as well as WHO standards compliance.

### Statistical analysis

The characteristics and results were reported as medians and ranges for each department. The two groups were compared by an external researcher, blinded to group allocation, using non-parametric statistics. Health status and standard compliance were analyzed using the Wilcoxon unpaired test, and service delivery frequencies were analyzed using Fisher’s exact test. *P* values below 0.05 were considered significant. All analyses were performed using SAS 9.4.

## Results

The response rate of departments was 40/48 (83%), but only 36/48 (75%) submitted complete data sets for the analyses. The data from the 36 departments covered 5285 patients (median per department = 147; range = 29–201) and 2529 staff members (70; 10–393). Overall missing data were 0–9% per factor (Table 1). All results were analyzed at department level.

### Health status of patients and staff

No differences in the health of patients or staff were found. At baseline, the intervention group’s (*n* = 22) SF36v2 patient PCS and MCS per department were 57 (11–95) and 61 (13–97). Their baseline staff PCS and MCS were 75 (36–97) and 71 (31–96).

**Fig. 2:**
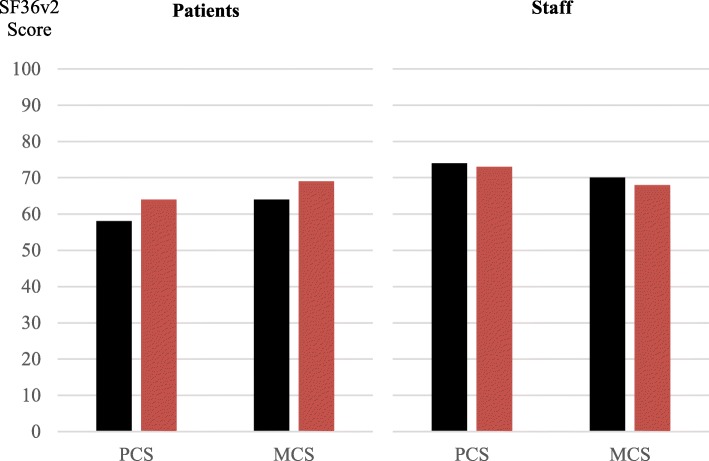
Comparison of the (*n* = 36) intervention (black) and control (red) departments’ patients (*n* = 5285. Median 147; range 29–201) and staff (*n* = 2529. 70; 10–393) health status by Short Form 36 version 2 (SF36v2) presented as median physical component score (PCS) and mental component score (MCS)

At follow-up, the intervention group patient PCS was 58 (7–96) versus 64 (12–98) in the control group (*p* = 0.19), and the patient MCS was 64 (9–98) versus 69 (17–99) (*p* = 0.25). The staff PCS was 74 (34–97) in the intervention group at follow-up versus 73 (36–97) (*p* = 0.58) in the control group, and the staff MCS was 70 (29–95) versus 68 (30–95) (*p* = 0.26). Figure 2 presents the PCS and MCS of patients and staff.

### Identification of lifestyle risks and related CHP service delivery

**Fig. 3:**
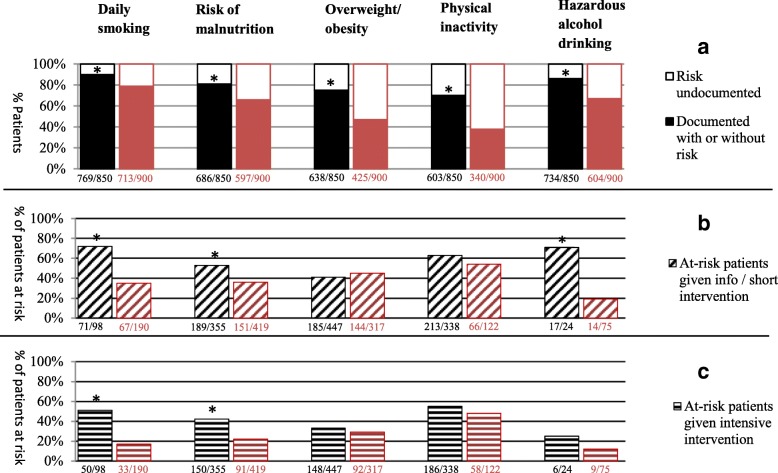
Comparison of the intervention (black) and control (red) departments’ documentation of their 1750 patients’ risks in the medical records (2a) and related delivery of information/shorter intervention (2b) and intensive intervention (2c) to at-risk patients (3% missing data. *P < 0.05)

The frequency of at-risk patients was similar: 252 (28%, 24–447) patients per risk factor on average in the intervention group versus 225 (26%, 75–419) in the control group. However, the completeness of patient risk documentation in the medical records was significantly better in the intervention group (81% versus 60%, *p* < 0.01). Delivery of information and/or short intervention (54% versus 39%, *p* < 0.01) and intensive intervention services (43% versus 25%, *p* < 0.01) to at-risk patients were also significantly more frequent in the intervention group. Figure 3 presents the completeness of the departments’ documentation of risk per factor and the degree to which systematic CHP interventions were then provided to documented at-risk patients.

Looking at the intervention group at baseline (*n* = 22), the completeness of risk documentation was 65%, delivery of information and/or short intervention services to at-risk patients was 40%, and delivery of intensive intervention services to at-risk patients was 35%.

### Standard compliance

**Fig. 4:**
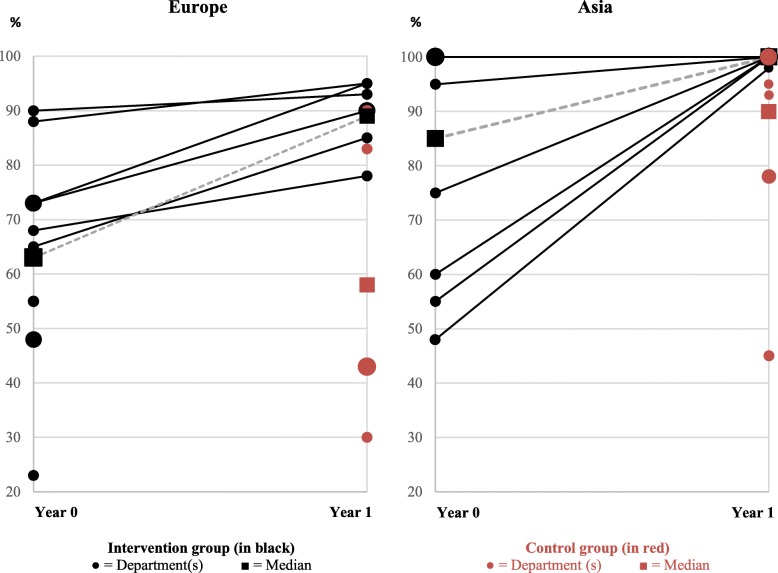
Compliance with WHO standards for health promotion in hospitals (in %) in the clinical departments of the intervention group (baseline *n* = 22, 1-year follow-up *n* = 18) and of the control group (*n* = 18)

The overall compliance with WHO standards was significantly higher in the intervention group (95% versus 80%, *p* = 0.02) (Table 2). Gold-level certificates for fulfilling ≥ 91% of standards were issued to 14 intervention group departments and 9 control group departments. Figure 4 shows the compliance improvements of the intervention group as well as the compliance of the control group. The in-group median standards compliance improvement within the intervention group was 12% (ranging 0–50%).

### Sensitivity analysis of the reported data

The reported WHO standards compliance and CHP service delivery were randomly evaluated during the site visits in the intervention group using external medical record audits and interviews. No significant differences were found (Table 3).

**Table 3:** Sensitivity analysis comparing the internal (IA) and external audit (at site visit) (EA) of medical records in the intervention group for documentation of four risk factors and related service delivery (IA = 850 medical records; in total 4 × 850 = 3.400. EA = 64 medical records; in total 4 × 64 = 256)

	Risk documentation			Service delivery to documented at-risk patients				
	IA	EA	*p*	IA		EA		*p*
	Documented/total (%)	Documented/total (%)		At-risk/documented (%)	Serviced risk/at-risk (%)	At-risk/documented (%)	Serviced risk/at-risk (%)	
Daily smoking	769/850 (90%)	61/64 (95%)		98/769 (13%)	57/98 (58%)	8/61 (13%)	4/8 (50%)	
Hazardous alcohol drinking	734/850 (86%)	55/64 (86%)		24/734 (3%)	13/24 (54%)	1/55 (2%)	0/1 (0%)	
Physical inactivity	603/850 (71%)	45/64 (70%)		338/603 (56%)	232/338 (69%)	35/45 (78%)	23/35 (66%)	
Nutritional problems	767/850 (90%)	62/64 (97%)		634/767 (83%)	283/634 (45%)	40/62 (65%)	23/40 (57%)	
Total (all four risk factors)	2873/3400 (85%)	223/256 (87%)	0.3	1094/2873 (38%)	585/1094 (53%)	84/223 (38%)	50/84 (60%)	*0.3*

### CHP service delivery to no-risk patients

Interestingly, CHP services were also delivered to patients with no or undocumented risks. Both groups provided CHP services to 13% of the documented no-risk patients and to 9–12% of patients with undocumented risks. In total, across all risks and regardless of risk documentation, 65% of patients in the intervention group and 47% in the control group received CHP services.

## Discussion

While we found no differences in the health status of patients and staff between groups, we did find that the intervention group identified patient lifestyle risks better and more frequently delivered related CHP services to at-risk patients compared to the control group that continued usual implementation routines. We also found that the intervention group had an overall higher compliance with the WHO standards.

### Service delivery to at-risk patients

Risk documentation and service delivery (Fig. 3) is relevant to healthcare quality, since these issues have been reported to be a general challenge [56, 57] not least for smoking [33, 58, 59]. Reported barriers include lacking treatment resources, awareness of the negative influence of lifestyle risks on treatment results, reimbursement of CHP services, management support, organizational focus on CHP, competencies of staff, and knowledge of the implementation process [33, 60, 61]. Suggested strategies to overcome these barriers include securing awareness of the evidence of CHP effectiveness, strengthening leadership engagement, and incentivizing CHP treatments [33].

Our results indicate that the operational program improved central parts of implementation within 1 year. The possible explanations for this improvement were explored in a nested qualitative study, and here, staff and managers echoed the already reported barriers and stated that the operational program increased awareness of and engagement in CHP within the departments [51]. In this light, it is plausible that the very presence of the study might have contributed to improved implementation by raising awareness of the implementation process. It is also possible that the prominent place CHP services have in the WHO standards may have contributed to improved implementation, and thus that the standards compliance increase found may in fact correlate with the improvements found for risk identification and service delivery. If this explanation holds, it is highly interesting that the Joint Commission has by now adopted tobacco and alcohol screening and treatment measures in their standards [62–64] and that the American College of Surgeons have added smoking as a risk factor in their National Surgical Quality Improvement Program [65]. The effects of adopting such risk documentation and CHP services will prove interesting in future.

### WHO standards compliance

Worldwide, healthcare systems use standards and indicators, and these are commonly viewed as an integral and justifiable part of quality management [66].

However, the evidence of the effect of quality improvement using standards and indicators is sparse [66–72]. Randomized studies have found effects on healthcare process and structure outcomes as a result of quality improvement programs [73–76], but either without investigating potential health effects [73, 76] or without finding effect on health [74, 75]. Non-randomized studies have shown mixed results; some have found health effects [77], while others have not [78]. On this basis, improving standards compliance alone (Table 2 and Fig. 4) is not sufficiently robust evidence to merit and inform action, and our study thus also included clinical outcomes related to actual risk identification, service delivery, and health status.

### Patient and staff health

For patients, the evidence of the positive effects of integrating CHP services in clinical treatments is growing [16, 17, 20, 21, 23, 24, 28, 33]. The reason we did not find evidence of health improvements among patients could relate to our following up on departments and not individuals. Thus, our study does not disprove health effects at the level of individual patients, which are expectable considering the effect of CHP interventions known from the literature on for instance smoking cessation [79]. In our study design, it is possible that fast flow of patients in the departments might have diluted health effects.

The WHO standards also include staff, and hospitals are notoriously hazardous workplaces [80]. Additionally, staff members are the ones delivering CHP to patients and both health and competencies of staff and managers have been shown to be associated with implementation of CHP. Smoking staff and managers, for instance, are less positive towards smoking cessation [81, 82]. Smoking staff less frequently deliver interventions [83] and follow-up services [84], and smoking managers less frequently adopt no smoking policies [82]. Lacking CHP competencies of staff are also a main barrier to actually delivering services [83, 84]. It seems probable then that improving both competencies and lifestyle risks among staff and managers might reduce barriers to CHP implementation.

The nested qualitative study, which was carried out alongside this RCT, reported that staff and managers were generally positive towards the operational program introduced and considered it to be worthwhile [51].

Just as for patients, the fact that we did not find an effect on staff health might be explainable by our following up on departments rather than individual staff members.

Even so, it can be noted that the staff in our study had a relatively high health-related quality of life, compared to the literature. The staff in our study had a PCS of 73–74 and an MCS of 68–70, as measured by SF36v2, whereas a 2013 sample of 2964 Norwegian nurses had lower PCS and MCS averages of 50 and 48, respectively, using the same 0–100 scale, but measured by SF12 [85].

Interestingly, staff and patients in our study had similar MCS. This could be due to a generally positive culture in HPH hospitals and because only three psychiatric departments took part. Compared to staff, patients had markedly lower PCS, which is readily explainable by their physical illness.

#### Bias and limitations

Our study has several biases and limitations. One major bias is that the study included only 48 of the targeted 80 departments. Of these, 40 (83%) departments participated, and 36 (75%) departments completed. This small number introduces a high risk of overlooking potentially significant results (e.g., health gain), which a fully powered study might have found (type-2 error).

Along with the open-call inclusion strategy, this might have resulted in a sample of departments with only highly motivated managements, potentially making our results optimistic. Likewise, the small sample size might also have meant that the randomization could not adequately minimize confounding differences between groups.

Another major limitation is the design that did not follow individual patients and staff, which was chosen due to our ambition to show a 1-year health effect at department level and thus avoid clustering. This alone might have rendered the study unable to identify health gains among patients and staff—provided such differences exist.

As this study is one of the first in its area, it might also suffer from risk of type-1 error regarding the significant results on risk identification, service delivery, and compliance. Further, sizeable studies would be needed to reduce this risk.

The lack of blinding resulting from the nature of the intervention adds further risk of bias. However, the analyses were blinded, and all reporting of results was performed in accordance with the level of randomization (i.e., department level), which avoided bias related to the use of clusters.

Both organizational and survey data were collected via self-assessment and self-reporting, which may introduce bias. However, these issues would presumably be relatively similar in both groups, since this is a ubiquitous type of bias in self-reported data, meaning that there is no apparent reason to speculate that it would be more present in one group than the other. Additionally, it was a strength that core tools used in the study had been validated in advance [37, 39, 40, 52, 53] and that the nested qualitative study indicated that staff generally found the project and its data collection doable [51].

Another risk of bias arose from the fact that most countries/regions participated with only a few hospitals each. This produced a skewed geographical distribution in both groups. However, the country stratification modified this risk, which was a strength.

The real-life conditions of the study were also a strength, but only HPH member hospitals were included, which potentially limits the generalization of our findings to non-HPH hospital settings. However, the international participation did broaden the representativeness of the results, which is a strength in terms of generalization.

It was a limitation that usual implementation routines naturally varied between participating departments, but it was a strength that site visits were performed and externally validated data from medical records.

Finally, it was a strength that the control group also showed improved service delivery and compliance after their 1-year implementation, when they were offered the intervention after the study had ended. In total, 17/18 control groups received the afterwards offered intervention, and their in-group results resembled those of the intervention group; standards compliance went up from 80 to 98%, documentation of risk from 60 to 85%, information/shorter intervention from 39 to 79%, and intensive intervention from 25 to 46%. As in the data for the study, no difference could be seen concerning health status.

#### Perspectives

Faster implementation of evidence has major implications in healthcare. In terms of the evidence for CHP, clinical departments and the healthcare system could potentially benefit from the operational program because it accelerates CHP implementation. In research, further investigation of the operational program in non-HPH settings should be undertaken. Also, use of the Fast-IM itself might turn out to accelerate evidence-based practices in other areas than CHP—e.g., new clinical procedures, new organizational improvements, or new technological initiatives or solutions.

A possible reason for the scarcity of RCTs in implementation as opposed to in intervention may be cultural and a result of research traditions, but it could also be that it is difficult to conduct them and recruit for them. Even so, interest does appear to be growing and the need for solid, experimental research and adequate reporting thereof is high [86]. We hope that such future, sizable trials will be able to draw from the learnings related to our operational program.

## Conclusion

Compared to usual implementation routines, the operational program improved implementation of CHP by better identification of lifestyle risks, more frequent delivery of CHP services, and higher compliance with standards. No differences in health status of patients or staff were found at the level of clinical departments, and the study was limited by low inclusion of departments and by having the department as the unit of analysis as opposed to individual patients and staff. These issues should be considered carefully in strategic implementation efforts and in designing new randomized studies.

## Additional file

Additional file 1: Project Materials. (PDF 426 kb)

## Abbreviations

CHP: Clinical health promotion
Fast-IM: The fast-track implementation model for clinical health promotion
HPH: International Network of Health Promoting Hospitals and Health Services
MCS: Mental component score (of SF36v2)
PCS: Physical component score (of SF36v2)
PDCA-cycle: Plan-Do-Check-Act Cycle for Quality Improvement
SF36v2: Short-Form 36 health questionnaire (version 2)
WHO: World Health Organization
WHO-CC Denmark: WHO Collaborating Centre for Evidence-based Health Promotion in Hospitals and Health Services
WHO-CC Sweden: WHO Collaborating Centre for Implementation of Evidence-based Clinical Health Promotion
