# Supplementary material for: A WHO-HPH operational program versus usual routines for implementing clinical health promotion: an RCT in health promoting hospitals (HPH)
Source: Implement Sci. 2018 Dec 22;13:153. doi: 10.1186/s13012-018-0848-0 (PMC6304000; doi:10.1186/s13012-018-0848-0)
Supplement: Supplementary file 1 — Project Materials. (PDF 426 kb) [file 13012_2018_848_MOESM1_ESM.pdf]

MANUAL (INTERVENTION GROUP)

In this HPH Recognition Project Participant Manual, we have explained each of the actions that you need to take one by one and in detail (as listed in the Project Time Line document). In this way, we hope it will be easy for you to know how to practically conduct the required actions.

The steps are:
1. Inclusion: Agreement and Allocation
2. Receiving Baseline Package
3. Data Collection: Internal Audit + Quality Plan
4. Returning Baseline Package
5. Implementation of Quality Plan
6. Receiving Follow Up Package
7. Data Collection; Internal Audit + Revised Quality Plan
8. Returning Follow Up Package
9. Data Validation: Site Visit + Certificate with results

1) Inclusion: Agreement and Allocation
This has been done by the Research Team in Copenhagen in accordance with the general guidelines for Randomized Clinical Trials. Upon inclusion you will receive a certificate of participation.
Your department has been allocated to the Intervention Group, which means you will begin the project immediately. It is a good idea to establish a relevant steering group at this early phase of the project. Please see the Quality Plan section below.

2 - 4) Baseline Package and Data Collection

<u>Receiving Baseline Package</u>
The Baseline Package contains everything you need to perform the first part of the Recognition Project in your department.
In the Baseline Package you will find:
- Welcome letter
- Project Timeline (specifically tailored for your department)
- Manual (showing you what to do and how to do it and giving good advice)
- Forms and web links for collecting data via:
    a. Internal MR Audit (patient health promotion)
    b. Patient Survey (patient health promotion)
    c. Staff Survey (staff health promotion)
    d. Organizational Data (organizational health promotion performance)
    e. Quality Plan (for making your quality plan)
- Project Protocol, describing the study in scientific detail (background information)

<u>The Internal Medical Record Audit (Enter data into Excel document)</u>
This is an audit of medical records from your department. It is performed by yourself or your staff.

The medical records you need to audit are:

MANUAL (INTERVENTION GROUP)

- 50 consecutive medical records, starting from the month prior to your department's inclusion in the project.
- This means that if your department joined the project on October 21, you should start your audit with medical records from the morning, at 8 o'clock, on September 21 – and continue until you have included the 50 consecutive medical records needed.
- You cannot exclude any record in any way. It is not relevant to include information or activities that your staff or you know from memory. You can only include the information that is clearly documented in the medical records.

In practice, the auditing of the records should be performed by two people. Usually, you would open the records and the data collection form (MR Audit Form). With this in hand the two people then go through the medical record and agree upon which documentation is there and which is not – all along ticking the relevant boxes in the form.

An example:

**WHO-HPH Recognition Project**<br>**MR Audit (Data collection sheet)**	**Department:** TW			**PA**
**Standard 2 – Patient assessment**		**1**	**2**	**3**
**2.2 Assessment of needs for HP (HPH DATA Model)**				
A1) Is the patient's BMI below 20.5?		▾		
A2) Has the patient lost weight in the past three months?		Y = Yes<br>N = No<br>U = Unknown		
A3) Has the patient had reduced appetite in the past week?				
A4) Is the patient severely ill? (i.e. stress-metabolic)				
B1) Is the patient's BMI above 25?				

If the documented information allows you to conclude that the patient's BMI is below 20.5 you should tick "yes". If it allows you to conclude that it is not below 20.5 you should tick "no". If no or insufficient information can be found in the record, you should tick "unknown". We ask that a fresh column (from 1 to 50) is filled out for each of the patients.

The Excel sheet you need is available in the Baseline Package, and we hope you will find it very simple to fill out.

Please add your allocation number in the top part of the Excel sheet, where it says "Department". Such as "TW103" for Taiwan, participating department number 103 or "CZ205" for Czech Republic participating department number 205.

<u>The Patient Survey (Online)</u>
The survey for patients should be filled out by the patient. If the patient needs support for doing this, a relative or a neutral staff member not involved in the project steering group may assist.

The patients you need to survey are:

MANUAL (INTERVENTION GROUP)

- In or out-patients that have entered your department from the day of the inclusion in the project and backwards one full month or until you have surveyed 200 consecutive patients (whichever comes first).
- This means that if your department joined the project on October 21, you should start surveying patients from morning, at 8 o'clock, on October 21 and backwards in time.
- You cannot exclude any patient, unless the patient has passed away, immigrated or is unable to give informed consent (unconscious, underage or so) or finally if the patient does not want to answer.

The link to your department's online survey forms are included in the Baseline Package. If needed, the survey may be printed out for the patient to fill in, and the answers subsequently entered into the online form by a neutral staff member.

Finally, please remember that all your survey forms should be numbered (at the very top of the form). When numbering, please use your allocation number plus a consecutive number for each patient (from P001 to P200). Such as "CZ205-P005" for Czech Republic participating department number 205, patient number 005.

If a patient does not respond as agreed, you are allowed to send out two reminders.

<u>The Staff Survey (Online)</u>
The survey for staff should be filled out by each staff member.

The staff members you need to survey are:

- All staff members employed at your department in the month prior to inclusion in the project.
- This means that if your department joined the project on October 21, you should survey staff that was employed at any given time in the calendar month of September (from the 1st to the 30th).
- You cannot exclude any staff member, unless he or she has passed away, immigrated or is unable to give informed consent or does not want to answer.

The link to your department's online staff survey forms are included in the Baseline Package. You need to give the staff the link to the survey and the password required – for instance by email.

Please remember that all your survey forms should be numbered (at the very top of the form). When numbering, please add your allocation number plus a consecutive number for each staff member (from S001 to S999). Such as "TW107-S010" for Taiwan, participating department number 107, staff member number 010.

If a staff member does not respond as agreed, you are allowed to send out two reminders.

MANUAL (INTERVENTION GROUP)

<u>The Organizational Data Form (pen and paper, signed copies of documentation, translation of highlighted passages)</u>

This is a paper form about how your department as an organization fulfils the administrative part of the WHO HPH Standards. There is just one document to fill out.

When filling out the form, all you need to do is:

- Tick the appropriate boxes, and
- wherever you tick “yes”, you then also make a copy of the material that is specified next to your “yes” in the column entitled “Documentation to deliver”.
- Remember to sign and date all copies of documentation

Note that if your documentation to deliver is not in English language already, you also need to:

- Highlight the passages in the copied material that document your tick-box answers – i.e. the passages that have to do with health promotion. Use a coloured highlighter pen.
- Translate these highlighted sentences into English. Please remember that you only need to translate the passages which you highlighted – no additional translation is required.
- Remember to sign and date all translations.

We have included the Organizational Data Form in the Baseline Package. Please be sure to number the form with your participating department number (such as US902, for USA, department 902). Likewise, please fill in the name of your department and the name of the person filling out the form.

Example:

1. Standard: Management Policy

The organization has a written policy for health promotion. The policy is implemented as part of the overall organization quality improvement system, aiming at improving health outcomes. This policy is aimed at patients, staff and community.

**1.1. The organization identifies responsibilities for health promotion.**	**Yes**	**No**	**Documentation to deliver**
1.1.1. The hospital's stated aims and mission include health promotion	X		Copy of material
1.1.2. Minutes of the governing body reaffirm agreement within the past year to participate in the WHO HPH project	X		Date for decision for annual fee payment
1.1.3. The hospital's current quality and business plans include health promotion (HP) for patients, staff and the community		X	Copy of action plan (highlighting HP)
1.1.4. The hospital identifies personnel and functions for the coordination of HP	X		Names of staff members that coordinate HP
**1.2. The organization allocates resources for the implementation of health promotion.**			
1.2.1. There is an identifiable budget for HP services [illegible]	X		Copy of budget or staff [illegible]

<u>Quality Plan</u>

MANUAL (INTERVENTION GROUP)

After gathering the required data in from MR Audit, Patient Survey, Staff Survey and Organizational Data Form, it is time to make your Quality Plan, based on the results. Please use the Quality Plan form.

- Describe why participation in the project is important and what can be achieved.
- Establish a multidisciplinary steering group. It is important to get both management and the different staff groups active and involved. It is the responsibility of the steering group to make a good and realistic quality plan.
- Analyse the collected data and describe the Baseline Status regarding health promotion in your department.
- Identify and discuss areas of well-established good practice and areas in need of development. This insight can be used in the next step.
- List the target areas you aim to improve upon throughout the project. Then describe what you will do to improve upon each of them, and which goals to meet.
- Make an implementation plan with clear milestones, follow ups and possibilities for adjustments.

5) Implementation of Quality Plan
When you have collected data, returned the Baseline and made your Quality Plan, it is time to implement it. You will have one full year to do that.

6 - 8) Follow Up Package
At the end of the implementation year, you will receive a Follow Up Package. This will be very similar to the Baseline Package and require you to repeat the data collections once again (in the exact same way as with the Baseline Package). The only difference will be that the Follow Up Package will entail revising the quality plan.
Concerning the data collection and revision of quality plan, please see above.

9) Data Validation, Site Visit and Certificate
The team in Copenhagen will make contact to schedule this with you, and provide detailed information in advance, after receiving the returned Follow Up Package.

Once your department has been successfully through the process, you will automatically be presented with a special certificate. The certificate will be the official HPH recognition of your achievements and it will display your level of performance (see figure below).

MANUAL (INTERVENTION GROUP)

1-25%	26-50%	51-70%	71-80%	81-90%	91-100%
Basic level	light green	green	bronze	silver	gold
○○○○○	●○○○○	●●○○○	●●●○○	●●●●○	●●●●●

Example: In this process, it is often beneficial to use the plan-do-check-act (PDCA) cycle, presented below.

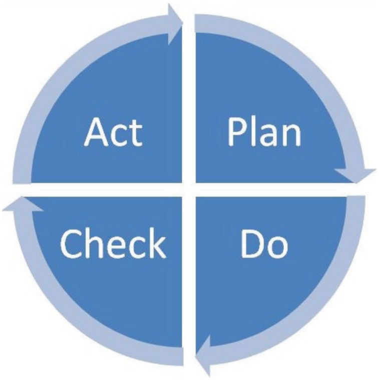

<u>Plan</u>: Planning the implementation of your quality plan. This entails filling out the Quality Plan form.
<u>Do:</u> Carrying out the Quality Plan and implementing the changes you decided upon. In this case for a period of one year.
<u>Check</u>: After the first year of the Project, it will be time to collect new results and analyse them. For this purpose you will receive a Follow-Up Package (very similar to the Baseline Package).
<u>Act:</u> Revise the Quality Plan according to new findings (and potentially, continue the cycle to keep improving).

Recognition Project

MR Audit (Data collection sheet)

Department:

PATIENTS 1 - 50 **Y** = Yes **N** = No **U** = Unknown

Standard 2 – Patient assessment	1	2	3	4	5	6	7	8	9	10	11	12	13	14	15	16	17	18	19	20	21	22	23	24	25	26	27	28	29	30	31	32	33	34	35	36	37	38	39	40	41	42	43	44	45	46	47	48	49	50
**2.2 Assessment of needs for HP (HPH DATA Model)**																																																		
A1) Is the patient's BMI below 20.5?																																																		
A2) Has the patient lost weight in the past three months?																																																		
A3) Has the patient had reduced appetite in the past week?																																																		
A4) Is the patient severely ill? (i.e. stress-metabolic)																																																		
B1) Is the patient's BMI above 25?																																																		
B2) Has the patient's waist exceeded 80 cm (W) or 94 cm (M)?																																																		
C1) Is the patient active less than 30 min/day? (Moderate intensity w. pulse increase, e.g. walking, cycling, training)																																																		
D1) Does the patient smoke daily?																																																		
E1) Does the patient's drinking exceed the recommend limits? (W= 7 per week, M= 14)																																																		
**2.3 Assessment of needs for HP (Additional information)**																																																		
Is there info from referring physician or others?																																																		
Do the social conditions indicate that the patient is at risk?																																																		
Does the religion require special diet or other specific attention?																																																		
**Standard 3 – Patient information and intervention**																																																		
**3.1 Based on HP needs, information given regarding**																																																		
Smoking																																																		
Hazardous alcohol intake																																																		
Nutritional problems																																																		
Physical inactivity																																																		
Psycho-social conditions																																																		
Others																																																		
Integrated																																																		
**3.1 Based on HP needs, motivational activities and brief intervention given regarding**																																																		
Smoking																																																		
Hazardous alcohol intake																																																		
Nutritional problems																																																		
Physical inactivity																																																		
Psycho-social conditions																																																		
Others																																																		
Integrated																																																		
**3.1 Based on HP needs, intervention, rehabilitation and after treatment given regarding**																																																		
Smoking																																																		
Hazardous alcohol intake																																																		
Nutritional problems																																																		
Physical inactivity																																																		
Psycho-social conditions																																																		
Others																																																		
Integrated																																																		
**Based on the above, follow-up for effect done regarding**																																																		
Smoking																																																		
Hazardous alcohol intake																																																		
Nutritional problems																																																		
Physical inactivity																																																		
Psycho-social conditions																																																		
Others																																																		
Integrated																																																		
**Standard 5 – Continuity and Cooperation**																																																		
**5. Assessment, rehabilitation and after treatment regarding**																																																		
Discharge summary including information of patients condition and health promotion needs																																																		
Discharge summary including information of HP internevntion																																																		
Plan for rehabilitation describing the role of organisation and partners																																																		

Patient survey

This survey asks for your views about your health. This information will help keep track of how you feel and how well you are able to do your usual activities.
The results will be handled in completely anonymized form by the International research group and the local quality group.
For each of the following questions, please tick the one box that best describes your answer.

1. Survey Form ID Number:

2. In general, would you say your health is:

- Excellent
- Very good
- Good
- Fair
- Poor

3. Compared to one year ago, how would you rate your health in general now?

- Much better now than one year ago
- Somewhat better now than one year ago
- About the same as one year ago
- Somewhat worse now than one year ago
- Much worse now than one year ago

4. The following questions are about activities you might do during a typical day. Does your health now limit you in these activities? If so, how much?

	Yes, limited a lot	Yes, limited a little	No, not limited at all
Vigorous activities, such as running, lifting heavy objects, participating in strenuous sports			
Moderate activities, such as moving a table, pushing a vacuum cleaner, bowling, or playing golf			
Lifting or carrying groceries			
Climbing several flights of stairs			
Climbing one flight of stairs			
Bending, kneeling, or stooping			
Walking more than a mile			
Walking several hundred yards			
Walking one hundred yards			

Bathing or dressing yourself			

5. During the past 4 weeks, how much of the time have you had any of the following problems with your work or other regular daily activities as a result of your physical health?

	All of the time	Most of the time	Some of the time	A little of the time	None of the time
Cut down on the amount of time you spent on work or other activities					
Accomplished less than you would like					
Were limited in the kind of work or other activities					
Had difficulty performing the work or other activities (for example, it took extra effort)					

6. During the past 4 weeks, how much of the time have you had any of the following problems with your work or other regular daily activities as a result of any emotional problems (such as feeling depressed or anxious)?

	All of the time	Most of the time	Some of the time	A little of the time	None of the time
Cut down on the amount of time you spent on work or other activities					
Accomplished less than you would like					
Did work or other activities less carefully than usual					

7. During the past 4 weeks, to what extent has your physical health or emotional problems interfered with your normal social activities with family, friends, neighbors, or groups?

- ☐ Not at all
- ☐ Slightly
- ☐ Moderately
- ☐ Quite a bit
- ☐ Extremely

8. How much bodily pain have you had during the past 4 weeks?

- ☐ None
- ☐ Very mild
- ☐ Mild
- ☐ Moderate
- ☐ Severe
- ☐ Very Severe

9. During the past 4 weeks, how much did pain interfere with your normal work (including both work outside the home and housework)?

- ☐ Not at all
- ☐ A little bit
- ☐ Moderately
- ☐ Quite a bit
- ☐ Extremely

10. These questions are about how you feel and how things have been with you during the past 4 weeks. For each question, please give the one answer that comes closest to the way you have been feeling. How much of the time during the past 4 weeks...

	All of the time	Most of the time	Some of the time	A little of the time	None of the time
Did you feel full of life?					
Have you been very nervous?					
Have you felt so down in the dumps that nothing could cheer you up?					
Have you felt calm and peaceful?					
Did you have a lot of energy?					
Have you felt downhearted and depressed?					
Did you feel worn out?					
Have you been happy?					
Did you feel tired?					

11. During the past 4 weeks, how much of the time has your physical health or emotional problems interfered with your social activities (like visiting friends, relatives, etc.)?

- ☐ All of the time
- ☐ Most of the time
- ☐ Some of the time
- ☐ A little of the time
- ☐ None of the time

12. How TRUE or FALSE is each of the following statements for you?

	Definitely true	Mostly true	Don't know	Mostly false	Definitely false
I seem to get sick a little easier than other					

people					
I am as healthy as anybody I know					
I expect my health to get worse					
My health is excellent					

On this page there are further questions about you, your lifestyle risks as well as the hospital department's services to you.

13. Gender

- Man
- Woman

14. Age

- 18 - 29
- 30 - 39
- 40 - 49
- 50 - 59
- 60 - 69
- 70 - 79
- 80 - 89
- 90 - 99
- 100 - 110

15. Are you aware of the health promotion policy of the hospital department?

- Yes
- No

16. How satisfied are you with the health promotion information given by the department?

- It was excellent
- It was very good
- It was good
- It was fair
- It was poor

17. When you left the department, were the follow-up health promotion instructions understandable?

- They were completely understandable

☐ They were mostly understandable

☐ They were partly understandable

☐ They were a little understandable

☐ They were not understandable

18. Has the department asked you about your health and lifestyle risks (such as smoking status)?

☐ Yes

☐ No

19. If you are a current or previous smoker have you received advice to quit smoking by the department?

☐ Yes

☐ Never smoker

☐ No

20. Has the department informed you of any supportive services, such as smoking cessation courses, which are available?

☐ Yes

☐ No

21. Please enter your

Height in cm: [ ]

Weight in kg: [ ]

22. Please enter your

Waist measurement in cm: [ ]

23. Have you lost weight in the past three months?

☐ Yes

☐ No

24. Has you had reduced appetite in the past week?

- [ ] Yes
- [ ] No

25. How many days per week are you physically active? (Moderate intensity with pulse increase, e.g. walking, cycling, training)

- [ ] 0
- [ ] 1
- [ ] 2
- [ ] 3
- [ ] 4
- [ ] 5
- [ ] 6
- [ ] 7

26. For the above days, how many minutes are you on average physically active?

Minutes (per day with physical activity): [ ]

27. How many days per week do you smoke/use tobacco?

- [ ] 0
- [ ] 1
- [ ] 2
- [ ] 3
- [ ] 4
- [ ] 5
- [ ] 6
- [ ] 7

28. For the above days, how many grams of tobacco do you smoke/use on average? (see gram conversion table below)

Grams of tobacco (per day with smoking/tobacco usage): [ ]

Tobacco gram conversion table:
1 cigarette = 1 gram
1 cheroot = 3 grams
1 cigar = 4 grams
1 pipe = 3 grams
1 snuff = 10 gram (nicotine content of 1 gram snuff corresponds to content of 10 cigarettes)

29. How many days per week do you drink alcohol?

- ☐ 0
- ☐ 1
- ☐ 2
- ☐ 3
- ☐ 4
- ☐ 5
- ☐ 6
- ☐ 7

30. For these days, how many units of alcohol do you drink on average? (see unit conversion table below)

Units of alcohol (per day with drinking): [ ]

Alcohol unit conversion table:

BEERS
1 light beer (33 cl., 1.5 – 2.5% vol) = 0.5 unit
1 small light beer (25 cl., 1.5 – 2.5% vol) = 0.25 unit
1 ordinary beer (33 cl., 3.5 – 4.5% vol) = 1 unit
1 small ordinary beer (25 cl., 3.5 - 4.5% vol) = 0.75 unit
1 strong beer (33 cl., 4.5 – 6% vol) = 1.25 units
1 small strong beer (25 cl. with 4.5 – 6% vol) = 1 unit
1 extra strong beer (33 cl., 7 – 10% vol) = 2 units
1 small extra strong beer (25 cl., 7 – 10% vol) = 1.25 units

WINES
1 glass of wine (12.5 cl., 11 - 16% vol) = 1 units
0.5 bottle of wine (37.5 cl., 11 - 16% vol) = 3.25 units
1 bottle of wine (75 cl., 11 - 16% vol) = 6.5 units

FORTIFIED WINES
1 glass of fortified wine (5 cl., 17 – 22% vol) = 0.5 unit

0.5 bottle of fortified wine (37.5 cl., 17 – 22% vol) = 5 units
1 bottle of fortified wine (75 cl., 17 – 22% vol) = 10 units

SPIRITS
1 ordinary serving of spirits (4 cl., 35 – 40% vol) = 1 unit
1 small serving of spirits (2 cl., 38% vol) = 0.5 unit
1 bottle of spirits (75 cl., 35 – 40% vol) = 19 units

OTHERS
1 RTD (Ready-To-Drink) or Alcopop (27.5 cl, 5.5% vol) = 1 units

31. If you find it difficult to calculate the exact number of drinks above, you are welcome to write your intake (per day with drinking) here (ex. half a bottle of red wine or half a 33 cl. bottle of strong beer etc.):

32. If you have any comments or reflections you want to share with the research group concerning this survey, you are welcome to write them here:

Staff Survey

This survey asks for your views about your health. This information will help keep track of how you feel and how well you are able to do your usual activities.
The results will be handled in completely anonymized form by the International research group and the local quality group.
For each of the following questions, please tick the one box that best describes your answer.

1. Survey Form ID Number:

☐

2. In general, would you say your health is:

☐ Excellent

☐ Very good

☐ Good

☐ Fair

☐ Poor

3. Compared to one year ago, how would you rate your health in general now?

☐ Much better now than one year ago

☐ Somewhat better now than one year ago

☐ About the same as one year ago

☐ Somewhat worse now than one year ago

☐ Much worse now than one year ago

4. The following questions are about activities you might do during a typical day. Does your health now limit you in these activities? If so, how much?

	Yes, limited a lot	Yes, limited a little	No, not limited at all
Vigorous activities, such as running, lifting heavy objects, participating in strenuous sports			
Moderate activities, such as moving a table, pushing a vacuum cleaner, bowling, or playing golf			
Lifting or carrying groceries			
Climbing several flights of stairs			
Climbing one flight of stairs			
Bending, kneeling, or stooping			
Walking more than a mile			
Walking several hundred yards			
Walking one hundred yards			

Bathing or dressing yourself			

5. During the past 4 weeks, how much of the time have you had any of the following problems with your work or other regular daily activities as a result of your physical health?

	All of the time	Most of the time	Some of the time	A little of the time	None of the time
Cut down on the amount of time you spent on work or other activities					
Accomplished less than you would like					
Were limited in the kind of work or other activities					
Had difficulty performing the work or other activities (for example, it took extra effort)					

6. During the past 4 weeks, how much of the time have you had any of the following problems with your work or other regular daily activities as a result of any emotional problems (such as feeling depressed or anxious)?

	All of the time	Most of the time	Some of the time	A little of the time	None of the time
Cut down on the amount of time you spent on work or other activities					
Accomplished less than you would like					
Did work or other activities less carefully than usual					

7. During the past 4 weeks, to what extent has your physical health or emotional problems interfered with your normal social activities with family, friends, neighbors, or groups?

- ☐ Not at all
- ☐ Slightly
- ☐ Moderately
- ☐ Quite a bit
- ☐ Extremely

8. How much bodily pain have you had during the past 4 weeks?

- ☐ None
- ☐ Very mild
- ☐ Mild
- ☐ Moderate
- ☐ Severe
- ☐ Very Severe

9. During the past 4 weeks, how much did pain interfere with your normal work (including both work outside the home and housework)?

- Not at all
- A little bit
- Moderately
- Quite a bit
- Extremely

10. These questions are about how you feel and how things have been with you during the past 4 weeks. For each question, please give the one answer that comes closest to the way you have been feeling. How much of the time during the past 4 weeks...

	All of the time	Most of the time	Some of the time	A little of the time	None of the time
Did you feel full of life?					
Have you been very nervous?					
Have you felt so down in the dumps that nothing could cheer you up?					
Have you felt calm and peaceful?					
Did you have a lot of energy?					
Have you felt downhearted and depressed?					
Did you feel worn out?					
Have you been happy?					
Did you feel tired?					

11. During the past 4 weeks, how much of the time has your physical health or emotional problems interfered with your social activities (like visiting friends, relatives, etc.)?

- All of the time
- Most of the time
- Some of the time
- A little of the time
- None of the time

12. How TRUE or FALSE is each of the following statements for you?

	Definitely true	Mostly true	Don't know	Mostly false	Definitely false
I seem to get sick a little easier than other					

people					
I am as healthy as anybody I know					
I expect my health to get worse					
My health is excellent					

On this page there are further questions about you, your lifestyle risks and your workplace.

13. Gender

- ☐ Man
- ☐ Woman

14. Age

- ☐ 18 - 29
- ☐ 30 - 39
- ☐ 40 - 49
- ☐ 50 - 59
- ☐ 60 - 69
- ☐ 70 - 79
- ☐ 80 - 89
- ☐ 90 - 99
- ☐ 100 – 110

15. Do you comply with the health and safety regulations of your workplace?

- ☐ I comply completely
- ☐ I mostly comply
- ☐ I comply partly
- ☐ I comply a little
- ☐ I don't comply at all

16. Are you aware of the risks at your workplace?

- ☐ I am completely aware
- ☐ I am mostly aware
- ☐ I am partly aware
- ☐ I am a little aware

☐ I am not at all aware

17. How do you rate your working conditions?

☐ Excellent

☐ Very good

☐ Good

☐ Fair

☐ Poor

18. Have you received introductory training, which addressed the health promotion policy of your workplace?

☐ Yes

☐ No

19. Are you aware of the content of the health promotion policy of your workplace?

☐ Yes

☐ No

20. Has your workplace asked you about your health and lifestyle risks (such as smoking status) within the last year?

☐ Yes

☐ No

21. Within the last year, has your workplace informed you of any supportive services, such as smoking cessation courses, which are offered to staff?

☐ Yes

☐ No

22. Please enter your

Height in cm: ____

Weight in kg: ☐

23. Please enter your

Waist measurement in cm: ☐

24. How many days per week are you physically active? (Moderate intensity with pulse increase, e.g. walking, cycling, training)

- ☐ 0
- ☐ 1
- ☐ 2
- ☐ 3
- ☐ 4
- ☐ 5
- ☐ 6
- ☐ 7

25. For the above days, how many minutes on average are you physically active?

Minutes (per day with physical activity): ☐

26. How many days per week do you smoke/use tobacco?

- ☐ 0
- ☐ 1
- ☐ 2
- ☐ 3
- ☐ 4
- ☐ 5
- ☐ 6
- ☐ 7

27. For the above days, how many grams of tobacco do you smoke/use on average? (see gram conversion table below)

Grams of tobacco (per day with smoking/tobacco usage): ☐

Tobacco gram conversion table:

1 cigarette = 1 gram
1 cheroot = 3 grams
1 cigar = 4 grams
1 pipe = 3 grams
1 snuff = 10 gram (nicotine content of 1 gram snuff corresponds to content of 10 cigarettes)

28. How many days per week do you drink alcohol?

- ☐ 0
- ☐ 1
- ☐ 2
- ☐ 3
- ☐ 4
- ☐ 5
- ☐ 6
- ☐ 7

29. For these days, how many units of alcohol do you drink on average? (see unit conversion table below)

Units of alcohol (per day with drinking): [ ]

Alcohol unit conversion table:

BEERS
1 light beer (33 cl., 1.5 – 2.5% vol) = 0.5 unit
1 small light beer (25 cl., 1.5 – 2.5% vol) = 0.25 unit
1 ordinary beer (33 cl., 3.5 – 4.5% vol) = 1 unit
1 small ordinary beer (25 cl., 3.5 - 4.5% vol) = 0.75 unit
1 strong beer (33 cl., 4.5 – 6% vol) = 1.25 units
1 small strong beer (25 cl. with 4.5 – 6% vol) = 1 unit
1 extra strong beer (33 cl., 7 – 10% vol) = 2 units
1 small extra strong beer (25 cl., 7 – 10% vol) = 1.25 units

WINES
1 glass of wine (12.5 cl., 11 - 16% vol) = 1 units
0.5 bottle of wine (37.5 cl., 11 - 16% vol) = 3.25 units
1 bottle of wine (75 cl., 11 - 16% vol) = 6.5 units

FORTIFIED WINES
1 glass of fortified wine (5 cl., 17 – 22% vol) = 0.5 unit

0.5 bottle of fortified wine (37.5 cl., 17 – 22% vol) = 5 units
1 bottle of fortified wine (75 cl., 17 – 22% vol) = 10 units

SPIRITS
1 ordinary serving of spirits (4 cl., 35 – 40% vol) = 1 unit
1 small serving of spirits (2 cl., 38% vol) = 0.5 unit
1 bottle of spirits (75 cl., 35 – 40% vol) = 19 units

OTHERS
1 RTD (Ready-To-Drink) or Alcopop (27.5 cl, 5.5% vol) = 1 units

30. If you find it difficult to calculate the exact number of drinks above, you are welcome to write your intake (per day with drinking) here (ex. half a bottled of red wine or half a 33 cl. bottle of strong beer etc.)

31. If you are a current or previous smoker have you received advice to quit smoking by your workplace?

- Yes
- No
- Never smoker

32. Have you ever been absent from your current job for 7 days or more in a row? (for any reason, except for holidays, pregnancy, education, planned leave etc.)

- Yes
- No

33. Have you ever been absent from your current job for 30 days or longer? (for any reason, except for holidays, pregnancy, education, planned leave etc.)

- Yes
- No

34. Within the last year, have you been injured at work? (including injuries such as needlestick, cuts from sharp devices etc.)

- Yes
- No

35. How burned-out from work are you?

- How burned-out from work are you? Not at all
- A little bit
- Moderately
- Quite a bit
- Extremely

36. With regards to patients with whom you have contact, how often are discharge letters sent on to the patient's GP or handed to the patient him- or herself?

- Very often or always
- Often
- Sometimes
- Once in a while
- Never
- I don't have patient contact

37. With regards to patients with whom you have contact, how often are they readmitted within 5 days of discharge?

- Very often
- Often
- Sometimes
- Once in a while
- Never
- I don't have patient contact

38. Upon discharge of patients with whom you have contact, how prepared are they to manage their own condition?

- They are completely prepared
- They are somewhat prepared
- They are partly prepared
- They are not at all prepared
- I don't have patient contact

39. If you have any comments or reflections you want to share with the research group concerning this survey, you are welcome to write them here:

ORGANIZATIONAL DATA FORM

Form ID:

Department:

Filled out by:

1. Standard: Management Policy<br><br>The organization has a written policy for health promotion. The policy is implemented as part of the overall organization quality improvement system, aiming at improving health outcomes. This policy is aimed at patients, staff and community.			
**1.1. The organization identifies responsibilities for health promotion.**	**Yes**	**No**	**Documentation to deliver**
1.1.1. The hospital's stated aims and mission include health promotion			Copy of material (highlighting HP)
1.1.2. Minutes of the governing body reaffirm agreement within the past year to participate in the WHO HPH project			Date for decision for annual fee payment
1.1.3. The hospital's current quality and business plans include health promotion (HP) for patients, staff and the community			Copy of action plan (highlighting HP)
1.1.4. The hospital identifies personnel and functions for the coordination of HP			Names of staff members that coordinate HP
**1.2. The organization allocates resources for the implementation of health promotion.**			
1.2.1. There is an identifiable budget for HP services and materials			Copy of budget or staff resources (highlighting HP)
1.2.2. Operational procedures such as clinical practice guidelines or pathways incorporating HP actions are available in clinical departments			Copy of guidelines (highlighting HP)
1.2.3. Specific structures and facilities required for health promotion (including resources, space, equipment) can be identified			Photos of structures and facilities (signed)
**1.3. The organization ensures the availability of procedures for collection and evaluation of data in order to monitor the quality of health promotion activities.**			
1.3.1. Data are routinely captured on HP interventions and available to staff for evaluation	Part of step 3 and 7 in the Recognition Project (staff survey)		
1.3.2. A programme for quality assessment of the health promoting activities is established			Copy of programme (highlighting HP)

ORGANIZATIONAL DATA FORM

2. Standard: Patient Assessment<br><br>The organization ensures that health professionals, in partnership with patients, systematically assess needs for health promotion activities.			
**2.1. The organization ensures the availability of procedures for all patients to assess their need for health promotion.**	**Yes**	**No**	**Documentation to deliver**
2.1.1. Guidelines on how to identify smoking status, alcohol consumption, nutritional status, psycho-social-economic status are present			Copy of guidelines
2.1.2. Guidelines / procedures have been revised within the last year			Date of revision and name of person that revised
2.1.3. Guidelines are present on how to identify needs for HP for groups of patients (e.g. asthma patients, diabetes patients, chronic obstructive pulmonary disease, surgery, rehabilitation)			Copy of guidelines (highlighting HP)
**2.2. The assessment of a patient's need for health promotion is done at first contact with the hospital. This is kept under review and adjusted as necessary according to changes in the patient's clinical condition or on request.**			
2.2.1. The assessment is documented in the patient's record at admission	Part of step 3 and 7 in the Recognition Project (MR Audit)		
2.2.2. There are guidelines / procedures for reassessing needs at discharge or end of a given intervention			Copy of guidelines
**2.3. The patient's needs-assessment reflects information provided by others and ensures sensitivity to social and cultural background.**			
2.3.1. Information from referring physician or other relevant sources is available in the patient's record	Part of step 3 and 7 in the Recognition Project (MR Audit)		
2.3.2. The patient's record documents social and cultural background as appropriate	Part of step 3 and 7 in the Recognition Project (MR Audit)		

ORGANIZATIONAL DATA FORM

3. Standard: Patient Information and Intervention<br><br>The organization provides patients with information on significant factors concerning their disease or health condition and health promotion interventions are established in all patient pathways.			
**3.1. Based on the health promotion needs assessment, the patient is informed of factors impacting on their health and, in partnership with the patient, a plan for relevant activities for health promotion is agreed.**	**Yes**	**No**	**Documentation to deliver**
3.1.1. Information given to the patient is recorded in the patient's record	Part of step 3 and 7 in the Recognition Project (MR Audit)		
3.1.2. Health promotion activities and expected results are documented and evaluated in the records	Part of step 3 and 7 in the Recognition Project (MR Audit)		
3.1.3. Patient satisfaction assessment of the information given is performed *(and the results are integrated into the quality management system)*	Part of step 3 and 7 in the Recognition Project (Patient survey)		
**3.2. The organization ensures that all patients, staff and visitors have access to general information on factors influencing health.**			
3.2.1. General health information is available			Copy of material and/or photos (signed)
3.2.2. Detailed information about high-risk diseases is available			Copy of material and/or photos (signed)
3.2.3. Information is available on patient organizations			Copy of material and/or photos (signed)

ORGANIZATIONAL DATA FORM

4. Standard: Promoting a Healthy Workplace<br><br>The management establishes conditions for development of the hospital as a healthy workplace.			
**4.1. The organization ensures the development and implementation of a healthy and safe workplace**	**Yes**	**No**	**Documentation to deliver**
4.1.1. Working conditions comply with national / regional directives and indicators			Copy of document recognising national and international regulations
4.1.2. Staff comply with health and safety requirements, all workplace risks are identified	Part of step 3 and 7 in the Recognition Project (staff survey)		
**4.2. The organization ensures the development and implementation of a comprehensive HR Strategy that includes training and development of health promotion skills of staff.**			
4.2.1. New staff receive an induction training that addresses the hospital's health promotion policy	Part of step 3 and 7 in the Recognition Project (staff survey)		
4.2.2. Staff in all departments are aware of the content of the organization's health promotion policy	Part of step 3 and 7 in the Recognition Project (staff survey)		
4.2.3. A performance appraisal system and continuing professional development incl. health promotion exists			Copy of material and number of appraisals conducted
4.2.4. Working practices (procedures and guidelines) are developed by multidisciplinary teams			Copy of practices (highlighting the team)
4.2.5. Staff are involved in hospital policy-making, audit and review			Copy of documents showing staff participation
**4.3. The organization ensures availability of procedures to develop and maintain staff awareness on health issues:**			
4.3.1. Policies for awareness on health issues are available for staff			Copy of policies on smoking, alcohol, drugs, physical activity and nutrition
4.3.2. Smoking cessation programmes are offered			Copy of programmes / photos (signed)
4.3.3. Annual staff surveys are carried out including an assessment of individual behaviour, knowledge on supportive services / policies, and use of supportive seminars	Part of step 3 and 7 in the Recognition Project (staff survey)		

ORGANIZATIONAL DATA FORM

5. Standard: Continuity and cooperation<br><br>The organization has a planned approach to collaboration with other health service providers and other institutions and sectors on an ongoing basis.			
**5.1. The organization ensures the health promotion services are coherent with current provisions and regional health policy plans**	**Yes**	**No**	**Documentation to deliver**
5.1.1. The management board is taking into account the regional health policy plan			Copy of document highlighting regulations and provisions
5.1.2. The management board can provide a list of health and social care providers working in partnership with the hospital			Copy of list
5.1.3. The intra- and intersectoral collaboration with others is based on execution of the regional health policy plan			Copy of document regarding congruency
5.1.4. There is a written plan for collaboration with partners to improve the patient's continuity of care			Copy of criteria for admittance and plan for discharge (highlighting HP)
**5.2. The organization ensures the availability and implementation of health promotion activities and procedures during out-patient visits and after patient discharge**			
5.2.1. Patients (and their families as appropriate) are given understandable follow-up instructions at out-patient consultation, referral or discharge	Part of step 3 and 7 in the Recognition Project (patient survey)		
5.2.2. There is an agreed upon procedure for information exchange practices between organizations for all relevant patient information			Copy of procedure
5.2.3. The receiving organization is given in timely manner a written summary of the patient's condition and health needs, and intervention provided by the referring organization	Part of step 3 and 7 in the Recognition Project (MR audit)		
5.2.4. If appropriate, a plan for rehabilitation describing the role of the organization and the cooperating partners is documented in the patient's record	Part of step 3 and 7 in the Recognition Project (MR audit)		

**Overall assessment of standards compliance**			
		Yes	No
1. Management Policy	Total 9		
2. Patient Assessment	Total 7		
3. Patient Information and Intervention	Total 6		
4. Promoting a Healthy Workplace	Total 10		
5. Continuity and Cooperation	Total 8		
**Overall**	**Total 40**	**/40**	**/40**

QUALITY PLAN

Department ID:

Department Name:

Background & Purpose
Patient-centred clinical health promotion has been shown to significantly improve both outcomes and patient safety. Accordingly, the WHO describes health promotion as a key dimension of quality in hospitals, and the organization has developed standards on the topic in order to help hospital management and staff members to assess and improve the quality of health care and thereby achieve better health for patients, staff and community.

Even so, however, health promotion is still a very implicit part of nearly all quality standards on hospitals. Moreover, assessing hospitals departments' health promotion performance is still quite an unexplored area.

On this basis, this project will test a new recognition process that uses the relevant WHO-HPH tools and standards to assess performance, by way of explicit documentation and evaluation of clinical health promotion activity.

The project is designed as an RCT, with an intervention group that undergoes the recognition process immediately and a control group that continue usual clinical routine. Then, after one year, the control group also begins the recognition process (= delayed start), while the Intervention group (=immediate start) continues with the recognition process.
This design allows for a great array of measurements, and the project thus aims to show whether the process really benefits implementation of health promotion in hospitals and health services, and also, if this, in the end, generates better health gains for patients and staff.

QUALITY PLAN

Importance for the Department

Here, please describe why participation in the project is important for the department

Multidisciplinary Steering Group Members

Name: ____________________
Function: Project Leader____________________

Name: ____________________
Function: ____________________

Name: ____________________
Function: ____________________

Name: ____________________
Function: ____________________

Name: ____________________
Function: ____________________

QUALITY PLAN

Baseline Status

Here, please use the main results from the data collection. We advice that your mark each item in colours (green, yellow and red) according to the inherent room for improvement, so that green items are areas that are ok already, yellow are areas in need of some improvement and red are areas in need of significant improvement.

<u>MR Audit Form</u>
Patient Assessment:
Patient Information and Intervention:
Continuity and Cooperation:

<u>Patient Survey</u>
Physical Health:
Mental Health:
Pain:
Health-Related Limitations:
Patient Satisfaction:
Follow-Up Instructions:
Awareness of Health Promotion Policy:

<u>Staff Survey</u>
Physical Health:
Mental Health:
Pain:
Health-Related Limitations:
Staff Satisfaction:
Awareness of Health Promotion Policy:
Safety, Risks and Work-Related Injuries:
Introductory Health Promotion Training:
Staff Assessment:
Absenteeism:
Burn-out:

<u>Organizational Data Form</u>
Management Policy:
Patient Assessment:
Patient Information and Intervention:
Promoting a Healthy Workplace:
Continuity and Cooperation:

QUALITY PLAN

**Target Areas**	**Goals**
Please list the target areas here<br>-<br>-<br>-<br>-<br>-<br>-	*Please list the related goals here*<br>-<br>-<br>-<br>-<br>-<br>-

Implementation Plan (12 months)

	**MONTHS (first year)**											
	**1**	**2**	**3**	**4**	**5**	**6**	**7**	**8**	**9**	**10**	**11**	**12**
**TARGET AREAS**												
-												
-												
-												
-												
-												
-												
